# Measurement of Secondary Products During Oxidation Reactions of Terpenes and Ozone Based on the PTR-MS Analysis: Effects of Coexistent Carbonyl Compounds

**DOI:** 10.3390/ijerph7113853

**Published:** 2010-11-01

**Authors:** Yusuke Ishizuka, Masahiro Tokumura, Atsushi Mizukoshi, Miyuki Noguchi, Yukio Yanagisawa

**Affiliations:** Department of Environment Systems, Institute of Environmental Studies, Graduate School of Frontier Sciences, The University of Tokyo, Kashiwa-no-ha 5-1-5, Kashiwa-shi, Chiba 277-8563, Japan; E-Mails: yusuke_ishizuka@yy.k.u-tokyo.ac.jp (Y.I.); atsushi_mizukoshi@yy.k.u-tokyo.ac.jp (A.M.); miyuki_noguchi@yy.k.u-tokyo.ac.jp (M.N.); yukio@k.u-tokyo.ac.jp (Y.Y.)

**Keywords:** terpene, ozone, secondary products, PTR-MS

## Abstract

Continuous measurements using proton transfer reaction mass spectrometry (PTR-MS) can be used to describe the production processes of secondary products during ozone induced oxidation of terpenes. Terpenes are emitted from woody building materials, and ozone is generated from ozone air purifiers and copy machines in indoor environments. Carbonyl compounds (CCs) are emitted by human activities such as smoking and drinking alcohol. Moreover, CCs are generated during ozone oxidation of terpenes. Therefore, coexistent CCs should affect the ozone oxidation. This study has focused on the measurement of secondary products during the ozone oxidation of terpenes based on the use of PTR-MS analysis and effects of coexistent CCs on oxidized products. Experiments were performed in a fluoroplastic bag containing α-pinene or limonene as terpenes, ozone and acetaldehyde or formaldehyde as coexistent CCs adjusted to predetermined concentrations. Continuous measurements by PTR-MS were conducted after mixing of terpenes, ozone and CCs, and time changes of volatile organic compounds (VOCs) concentrations were monitored. Results showed that, high-molecular weight intermediates disappeared gradually with elapsed time, though the production of high-molecular weight intermediates was observed at the beginning. This phenomenon suggested that the ozone oxidation of terpenes generated ultrafine particles. Coexistent CCs affected the ozone oxidation of α-pinene more than limonene.

## 1. Introduction

Ozone is transported from outdoor environments to indoor environments by ventilation, and indoor ozone concentrations are typically about 20–70% of concurrent outdoor levels [1]. Outdoor ozone concentrations reach at maximum 6.12 × 10^−2^–2.04 × 10^−1^ ppm (120–400 μg m^−3^) [2]. Additionally, air cleaners using ozone have been widely used in residences in recent years, and they have been believed indoor sources of ozone [3–5]. Some photocopiers and printers can also generate ozone [6–8]. According to the literature [9], ozone concentrations in poorly ventilated rooms which contained electrostatic air cleaners and photocopying machines were up to 2.50 × 10^−1^ ppm (490 μg m^−3^). Therefore, ozone concentrations in residences are believed higher than estimated from outdoor ozone concentration and indoor ozone concentrations should be especially high around printers, photocopiers and air purifiers [10,11].

Terpenes are emitted from woody building materials [12,13], which are the materials of choice in Japanese residences. Because terpenes have pleasant smells, they are used extensively as ingredients in many household products, such as air fresheners [12–15]. For example, a limonene concentration as high as 1.75 × 10^−1^ ppm (975 μg m^−3^) was measured after applying a spray wax to a coffee table [16] and around 3.50 × 10^−1^ ppm (1950 μg m^−3^) when peeling an orange [17]. Additionally, terpenes are also widely used in solvents, paintings, deodorants and varnishes [12,13]. From these reasons, terpene concentrations can often increase to significant levels in poorly ventilated rooms [18].

Carbonyl compounds (CCs) are emitted in homes as a result of occupants’ activities, and acetaldehyde in particular is measured in high concentrations during smoking [19] and alcohol drinking [20]. Additionally, formaldehyde and acetaldehyde are generated during the terpene oxidation [14,21].

Terpenes have one or more carbon-carbon double bonds in their molecular structures. Due to this, terpenes have a high oxidation activity towards oxidants such as ozone, nitroxide and hydroxyl (OH) radicals in the atmosphere [21,22]. For instance, reactions of ozone and terpenes lead to the production of carbonyls such as formaldehyde and acetaldehyde, organic acids, hydrogen peroxide, secondary organic aerosols and OH radicals [14,21]. Produced OH radicals would chain-react with volatile organic compounds (VOCs) or CCs in indoor environments, resulting in the generation of additional oxidation products [8]. Although a large volume of research related to the production of secondary organic aerosols has been reported [15,23–25], information about many of the individual secondary products during ozone oxidation of terpenes is limited [8]. Additionally, the mixture of oxidation products appears to have significant irritant properties [26].

On the other hand, coexistent CCs in residential indoor environments should affect the oxidation reaction products. However, only a few studies have been reported on effects of coexistent CCs in residential indoor environments on oxidation reaction products [24,28]. This study focuses on the effects of the incorporation of coexistent CCs in residential indoor environments into ozone-terpenes oxidation reactions based on proton transfer reaction mass spectrometry (PTR-MS) analysis. α-Pinene and limonene were used as model terpenes, and acetaldehyde and formaldehyde were used as model coexistent CCs in residential indoor environments. In residential indoor environments, the aldehydes are emitted from furniture made of particleboards and produced with urea-formaldehyde resins [29–31].

In this study, secondary products during the ozone oxidation of terpenes were monitored using the PTR-MS technique. The PTR-MS is an analytical instrument for online measurements of trace amount of VOCs, including oxygenated VOCs such as acetaldehyde and terpene oxidation products. The PTR-MS system has a high sensitivity to VOCs and allows direct air inlet and real-time analysis.

## 2. Experimental Section

### 2.1. Materials

α-Pinene of 98%, limonene [(R)-(+)-limonene] of 97% and formaldehyde (paraformaldehyde) of 95% purity were obtained from Sigma-Aldrich Japan. Acetaldehyde of 90% purity was purchased from Wako Pure Chemical Industries, Ltd., Japan. Pure air was supplied by Japan Air Gases Co. They were all used without any further purification.

### 2.2. Methods

Gases of α-pinene and limonene were prepared by evaporation of liquids in a 1 L air sampling tube (GL Sciences, Japan). Acetaldehyde gas was generated by a gas generator (PD-1B, GASTEC Corporation, Japan) with a permeation tube (GASTEC Corporation, Japan). Formaldehyde gas was generated by the gas generator and prepared from paraformaldehyde which was put in a 2 mL vial (SUPELCO, Japan) equipped with a glass filter paper (Whatman, Japan). In order to adjust the desired concentrations of organic gases, these were introduced into a 5 L fluoroplastic bag and diluted with pure air. Ozone was generated by a UV light ozone generator (SO-100, FUNATECH CO., Japan) which generates no NOx during the ozone generation. The adjustment of ozone concentration was carried out in a 2 L fluoroplastic bag using the pure gas. Relative humidities of adjusted organic gases were less than 15% measured by a HOBO data logger (U10, Onset Computer Corporation).

Organic gases and ozone were introduced into the 2 L fluoroplastic sample bag in a dark temperature-controlled room (24.5 ± 0.3 °C), and then ozone-terpenes oxidation reactions were started. During the oxidation reaction, samples from the gas phase were withdrawn at predetermined time intervals using a control valve, and organic gas concentrations including secondary products were analyzed by on the PTR-MS instrument (Ionicon Analytik GmbH, Austria). The mass range analyzed in this study was from *m*/*z* 21 to 220.

The PTR-MS is a quadrupole mass spectrometer that uses hydronium ions (H_3_O^+^) to chemically ionize the compound of interest through a proton transfer reaction. Thus, any compound that has a proton affinity higher than that of water can be detected, and is identified by their molecular weight plus 1 (H^+^) peaks. The PTR-MS can detect highly polar molecules such as oxidized organic compounds, and its short accumulation times (the accumulated time is 50 ms per each mass number) allows real-time measurements [23,24]. Although peak signal intensity relates to absolute concentration of the compound related to its *m*/*z* value, it does not exhibit accurate concentration due to fragmentation. Therefore, calibration curves for organic gases made from preliminary experimental results using GC/MS and HPLC were used. Experimental conditions used in this study are listed in Table 1. Each experiment was repeated three times (*n* = 3).

## 3. Results and Discussion

### 3.1. Decomposition Rates of Terpenes by Reaction with Ozone

**Figure 1:**
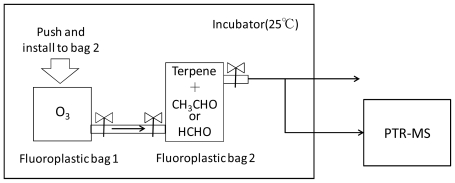
Apparatus for ozone oxidation of terpenes.

In order to estimate effects of coexistent CCs on the rates of degradation of α-pinene and limonene by ozone, time changes of concentrations of α-pinene (runs No. 1 to 5) and limonene (runs No. 6 to 10) during the ozone oxidation of terpenes in the presence or absence of coexistent CCs are shown in Figure 1. As it can be seen from Figures 1a and 1b, concentrations of α-pinene and limonene decreased with elapsed time because the α-pinene and limonene were decomposed by the reaction with ozone [21,23,24,32–34]. Decompositions of 90% α-pinene and limonene were achieved within 8 min and 4 min, respectively, regardless of coexistent CCs. Limonene exhibited higher reactivity towards ozone than α-pinene. The reason of this tendency will be discussed later.

According to literatures [32,33], the degradations of α-pinene and limonene by ozone obey the second order kinetics with respect to the concentrations of terpenes and ozone expressed in the following equations:

(1) $$\frac{{dC}_{\alpha }}{dt}=-k_{\alpha }C_{O3}C_{\alpha }$$

(2) $$\frac{{dC}_{L}}{dt}=-k_{L}C_{O3}C_{L}$$

where *C*_α_ is an α-pinene concentration, *C*_L_ is a limonene concentration, *C*_O3_ is an ozone concentration, *k*_α_ and *k*_L_ is second order kinetic constants and *t* is a elapsed time.

**Figure 2:**
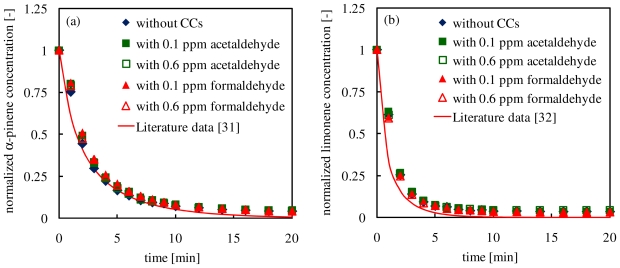
Time changes of concentrations of (a) α-pinene and (b) limonene during the ozone-terpenes oxidation reactions.

In the literature [35,36], *k*_α_ and *k*_L_ were determined as 8.11 × 10^−17^ and 2.01 × 10^−16^ cm^3^ molecule^−1^ s^−1^, respectively. Solid lines in Figures 2a and 2b show predictions based on these reaction rate constants. It was found that the experimental result of the α-pinene ozonolysis was in good agreement with the prediction based on the literature data, regardless of coexistent CCs.

On the other hand, the experimental results of ozone-limonene oxidation showed a slightly slower degradation rate than that predicted based on the literature data. This may be attributed to the fact that because these reaction rate constants were determined as reaction rate constants of individual reactions, predictions based on the literature data did not take into account for reactions of ozone with intermediates. In the α-pinene degradation experiments, α-pinene has one unsaturated bond in its molecular structure, which would be decomposed by the attack of electrophilic ozone. Therefore, intermediates produced during the ozone oxidation of α-pinene would not have unsaturated bonds in their molecular structure, which would lead to a lower reactivity of the ozone due to the decrease in the electron density. Because of the reactivity difference between α-pinene and intermediates, ozone was consumed predominantly by the oxidation of α-pinene, and the presence of intermediates can be ignored.

Meanwhile, limonene has two unsaturated bonds in its molecular structure, therefore, intermediates produced during the ozone oxidation of limonene would still have one unsaturated bond in their molecular structures, and can react with ozone strongly. This would lead to the competition between limonene ozonolysis and reactions of intermediates with ozone. As a result, the limonene ozonolysis was only slightly inhibited, and there was little disagreement between the experimental data and the literature data.

In order to compare degradation rates quantitatively, overall pseudo second order kinetic constants which exclude reactions of intermediates with ozone were determined by fitting the experimental data to the kinetic model predictions using a least-squares fitting technique. Results are shown in Table 2.

According to the results of preliminary experiments which evaluated reactions of acetaldehyde or formaldehyde with ozone, concentrations of acetaldehyde and formaldehyde were almost constant until 60 min. According to the literature [35,36], the second order kinetic constants of acetaldehyde and formaldehyde are 3.4 × 10^−20^ and 2.09 × 10^−24^ cm^3^ molecule^−1^ s^−1^, respectively. Compared to the second order kinetic constants of α-pinene and limonene (8.11 × 10^−17^ and 2.01 × 10^−16^ cm^3^ molecule^−1^ s^−1^), the second order kinetic constants of acetaldehyde and formaldehyde are 10^3^ times to 10^8^ times smaller. Therefore, reactions of acetaldehyde and formaldehyde with ozone can be ignored. As shown in Table 2, the degradation rates of α-pinene and limonene were not affected by coexistent CCs, because, even if acetaldehyde or formaldehyde exist, the ozone would react preferentially with α-pinene or limonene.

### 3.2. Effects of Incorporation of CCs on Time Changes of Acetaldehyde and Formaldehyde Concentrations

**Figure 3:**
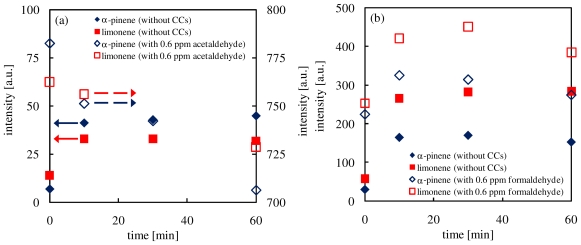
Time changes of concentrations of (**a**) acetaldehyde (*m*/*z* 45) and (**b**) formaldehyde (*m*/*z* 31) during the ozone-terpenes oxidation reactions.

In the PTR-MS analysis, the ion signals at *m*/*z* 45 and 31 mainly indicate acetaldehyde and formaldehyde, respectively. Carbon dioxide has the same mass as acetaldehyde, but it has low proton affinity and is not detected by the PTR-MS analysis [33]. Figure 3 shows temporal changes of concentrations of acetaldehyde (*m*/*z* 45) (a) and formaldehyde (*m*/*z* 31) (b) during the ozone-terpenes oxidation reactions.

It can be seen from Figure 3, acetaldehyde and formaldehyde were produced during the terpene ozonolysis. Particularly, significant amounts of formaldehyde were produced. In the ozone oxidation of terpenes, OH radicals are produced as byproducts [34]. The reactions of terpenes and OH radicals leads to the generation of methyl radicals [38], which subsequently react with oxygen, and formaldehyde is produced [38].

As mentioned before, acetaldehyde and formaldehyde were not decomposed by ozone. However, when acetaldehyde or formaldehyde was added into the ozone-terpene reactions, their concentrations decreased slightly, as shown in Figure 3. Particularly, the acetaldehyde concentration decreased with time significantly. These phenomena suggested that acetaldehyde or formaldehyde bound to intermediates produced by the ozone oxidation of terpenes. Therefore, the addition of acetaldehyde would affect the production of secondary compounds.

### 3.3. PTR-MS Spectra during the Reaction of Terpenes with Ozone

**Figure 4:**
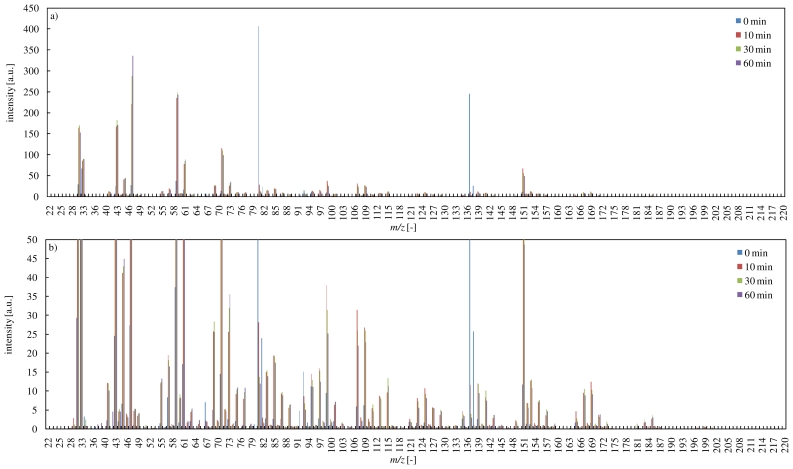
PTR-MS spectra during the reaction of α-pinene with ozone (a: overall view, b: magnified figure).

PTR-MS spectra during the ozone oxidation of α-pinene are shown in Figure 4. The PTR-MS ion signals at *m/z* 81, 82, 137 and 138 represent the α-pinene fragments, isotopes and their protonated ions, respectively [21]. After 10 min, many signals continued to change, as shown in Figure 4, though α-pinene was decomposed completely as shown in Figure 2. This indicated that secondary ozonides would be produced over a long period. According to the literature [34], α-pinene oxide (*m/z* 153), isopropylideneacetone (*m/z* 99), (2,2-dimethyl-3-acetylcyclobutyl)methyl formate, (acetyl-2,2,3-trimethyl)-cyclobutane (*m/z* 185), pinonic acid (*m/z* 186), norpinonaldehyde (*m/z* 155), 10-hydroxyl-pinonic acid (*m/z* 201) and (2,2-dimethylcyclobutyl)acetaldehyde (*m/z* 127) would be produced as secondary ozonides during the ozone oxidation of α-pinene. In this study, PTR-MS ion signals represented these secondary ozonides, and they were in good agreement with the literature. The ozone oxidation of α-pinene caused the production of secondary ozonides which have high molecular weight, as described in Figure 4. Particularly, verbenone (*m/z* 151) was produced in relatively large amounts.

**Figure 5:**
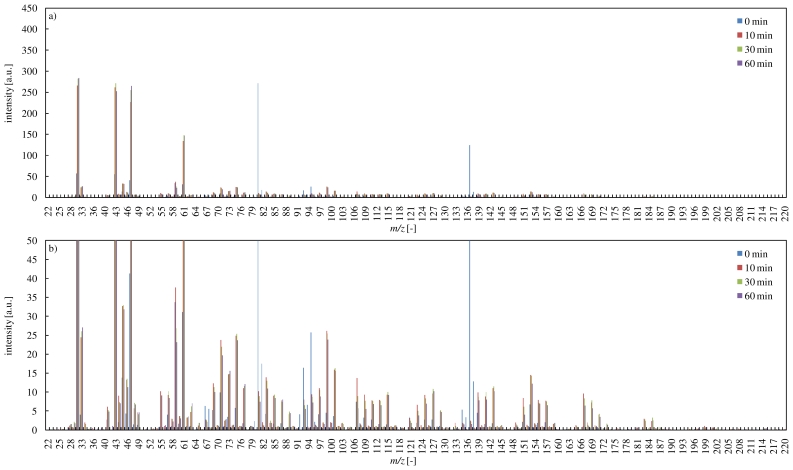
PTR-MS spectra during the reaction of limonene with ozone (a: overall view, b: magnified figure).

Figure 5 shows PTR-MS spectra during the reaction of limonene with ozone. In the reaction, a large number of secondary ozonides were generated, as indicated by the many signals. However, signal intensities in the reaction of limonene were smaller compared with the reaction of α-pinene with ozone. This might be attributed to the difference of reactivities with ozone and primary intermediates. Primary intermediates produced in the reaction of limonene with ozone have an unsaturated bonds, and would exhibit higher reactivity, as mentioned before. Therefore, these primary intermediates would be decomposed by ozone early. On the other hand, primary intermediates produced in the reaction of α-pinene with ozone have no unsaturated bonds, and might remain for a relatively-long term due to their low reactivity. These primary intermediates might be attacked by radicals produced in the reaction of terpenes with ozone. As a result, a large number of higher molecular weight secondary products would be produced in the reaction of terpenes with ozone.

### 3.4. Effects of Coexistent CCs on the Reaction of Terpenes and Ozone

**Figure 6:**
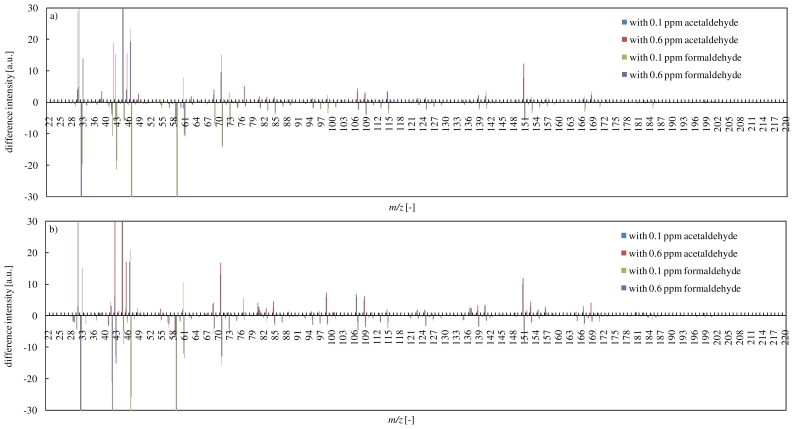
Effects of coexistent CCs on the reaction of α-pinene with ozone (a: at 10 min of elapsed time, b: at 60 min of elapsed time).

In order to examine effects of coexistent CCs on the reaction of α-pinene with ozone, differences of PTR-MS spectra with or without coexistence CCs at 10 min and 60 min are shown in Figure 6. In Figure 6, difference intensity means a difference between intensities obtained in experiments with and without coexistence CCs (“difference intensity” = “intensity under coexistence of CCs” − “intensity under no-coexistence of CCs”). Positive values of difference intensity can reflect production enhancement, and then negative values can reflect production inhibition.

It was found clearly that the addition of acetaldehyde as coexistent CCs into the reaction of α-pinene with ozone indicated many positive values of difference intensity, which tended to increase types of secondary products. In other words, the decomposition pathway of α-pinene by the ozone oxidation was affected by the addition of acetaldehyde. Even in the low acetaldehyde concentration (0.1 ppm), the decomposition pathway was affected. Although the decomposition of α-pinene was mostly finished by 10 min, secondary products productions were still continued. In particular, secondary products which have a high molecular weight (e.g., *m/z* 167 and 169) were produced after 60 min. This phenomenon suggested that the addition of acetaldehyde led to the long term production of higher molecular weight secondary products. On the other hand, the addition of formaldehyde clearly inhibited secondary products productions. This result might be attributed to the production of stable intermediates. Although both of acetaldehyde and formaldehyde are classified into carbonyl compounds, they showed the opposite effect on the productions of secondary products. These phenomena suggested that coexistence of CCs have made the ozone oxidation of terpenes in another reaction pathway progressed.

**Figure 7:**
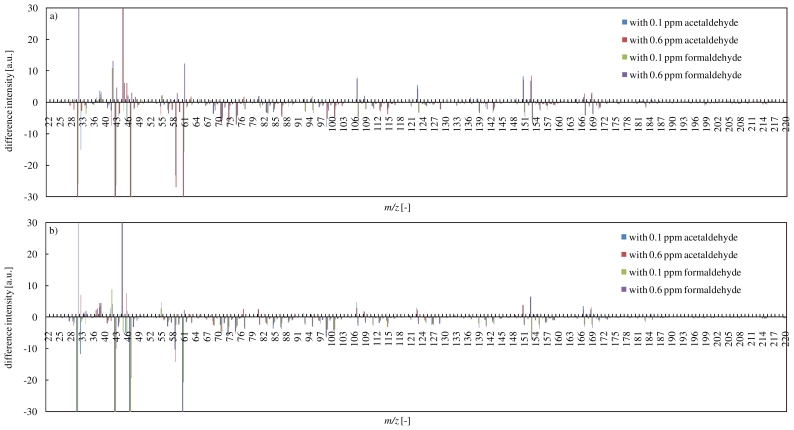
Effects of coexistent CCs on the reaction of limonene with ozone (a: at 10 min of elapsed time, b: at 60 min of elapsed time).

Effects of coexistent CCs on the reaction of limonene with ozone are shown in Figure 7. The addition of acetaldehyde enhanced secondary products productions such as *m/z* 151, 153, 107. However, unlike the case in the reaction of α-pinene, this also inhibited the production of secondary products such as *m/z* 99, 101, 71, 73, 75.

### 3.5. Effects of Added CCs on Condensation

In the PTR-MS analysis, values of *m/z* and intensity of peak signals are correlated to mass number and concentration, respectively. In this study, the summation of multiplying each value of *m/z* by the intensity is determined as total mass amount (TMA) of secondary products (Equation (3)).

(3) $$total\;mass\;amount\;(TMA)=\sum I_{i}m_{i}/z_{i}$$

During the ozone oxidation, because of an addition of oxygen atoms to terpenes or secondary products, TMA would increase with elapsed time. On the other hand, TMA would decrease by a production of intermediates which cannot be detected by the PTR-MS analysis such as CO_2_ and N_2_O [35].

However, according to preliminary experimental results, ozone cannot degrade acetaldehyde and formaldehyde which are among the final intermediates after 60 min. Therefore, under these experimental conditions, TMA cannot decrease except for the production of high molecular weight secondary products (*m/z* > 220) by combination or if condensation onto secondary organic aerosols occurred. Consequently, the decreases in TMA can mainly indicate a condensation tendency.

**Figure 8:**
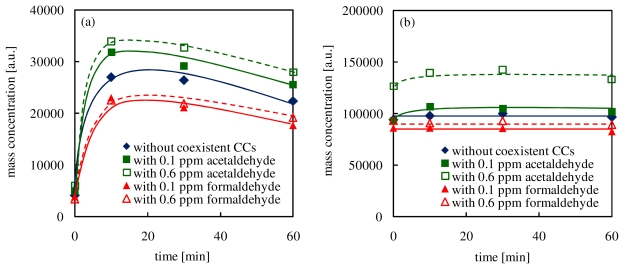
Effects of coexistent CCs on the combination in the reaction of α-pinene with ozone (TMA in the mass range (a) above *m*/*z* 139 and (b) below *m*/*z* 138).

In order to investigate effects of the addition of CCs on the condensation in the reaction of α-pinene with ozone, time changes of TMA (mass range: less than *m*/*z* 138 and more than *m*/*z* 139) are shown in Figure 8. In this study, because mass numbers of derived α-pinene are *m/z* 81, 82, 137 and 138, the TMA increases in the mass range above *m*/*z* 139 are attributed to combination with oxygen or intermediates.

**Figure 9:**
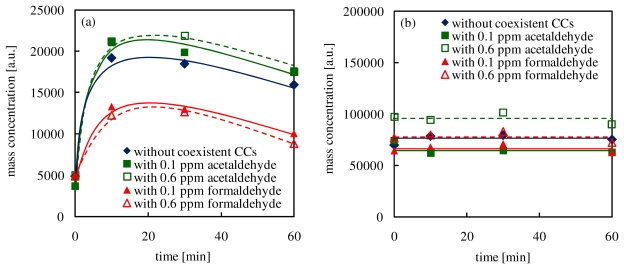
Effects of coexistent CCs on the combination in the reaction of limonene with ozone (TMA in the mass range (a) and above *m*/*z* 139 (b) below *m*/*z* 138).

As shown in Figure 8a, TMA in the mass range above *m*/*z* 139 increased significantly at the beginning of the reaction. While TMA increments when acetaldehyde was added into the reaction of α-pinene with ozone were larger than the experiments without coexistent CCs, the addition of formaldehyde decreased TMA increses. These results suggested that the addition of acetaldehyde activates the production of secondary products having a high reactivity in the reaction of α-pinene with ozone, which might ultimately activate the generation of secondary organic aerosols. Conversely, the addition of formaldehyde inhibited TMA increases. This result indicated that the production of stable secondary products in the early stage was improved by the addition of formaldehyde. After 10 min of elapsed time, TMA in the mass range above *m*/*z* 139 decreased with time. On the other hand, TMA in the mass range below *m*/*z* 138 in all cases was almost constant after 10 min. If decompositions of secondary products having a mass number above m/z 139 occurred, TMA in the mass range below *m*/*z* 138 would increase due to the production of lower molecular weight compounds. However, TMA in the mass range above *m*/*z* 139 increased with no increases in TMA in the mass range under *m*/*z* 138. It seems that high molecular weight secondary products (*m/z* > 220) were produced by combination and they were condensed onto secondary organic aerosols. According to the literature [39], secondary products are produced by reactions of high reactive secondary products and acetaldehyde exhibit a low boiling point, which causes condensation of secondary products. These results show good agreement with the results in this study. Figure 9 shows effects of coexistent CCs on combination in the reaction of limonene with ozone. Like the results in the experiments with α-pinene, the addition of acetaldehyde improved the polymerization, and the incorporation of formaldehyde inhibited it.

## 4. Conclusions

Effects of coexistent CCs on the ozone oxidation of terpenes were investigated by the measurement of secondary products during the ozone oxidation of terpenes based on PTR-MS analysis. The addition of coexistent CCs clearly affected the reaction pathways of the ozone oxidation of terpenes, even in the case of low concentrations of coexistent CCs. Secondary products were significantly affected by the type of compound used. Although both acetaldehyde and formaldehyde are classified as carbonyl compounds, they showed opposite effects on the productions of secondary products. The mechanisms of the influences of the addition of coexistent CCs are not clear in this study, because the ozone oxidation of terpenes involves many complex reactions. However, this research did suggest qualitatively the effects of the addition of coexistent CCs on the ozone oxidation of terpenes based on the PTR-MS analysis. That is, the addition of acetaldehyde activated the production of high molecular weight secondary products, and the addition of formaldehyde inhibited the production of high molecular weight secondary products during the ozone oxidations of terpenes.

Experimental results in this study could help to reveal the accurate mechanism for the production of secondary organic aerosols by the ozone oxidation of terpenes. In order to determine the reaction pathway in the reactions of terpenes with ozone with coexistent CCs, secondary products should be identified in future work. To understand of effects of coexistent CCs is important to make residential indoor environments safer.

## Figures and Tables

**Table 1:** Conducted experimental conditions (*n* = 3).

Run NO.	Terpene concentrations	CCs concentrations	Ozone concentration
1	3 ppm (α-pinene)	-	4.5 ppm
2		0.1 ppm (Acetaldehyde)	
3		0.6 ppm (Acetaldehyde)	
4		0.1 ppm (Formaldehyde)	
5		0.6 ppm (Formaldehyde)	
6	3 ppm (limonene)	-	
7		0.1 ppm (Acetaldehyde)	
8		0.6 ppm (Acetaldehyde)	
9		0.1 ppm (Formaldehyde)	
10		0.6 ppm (Formaldehyde)	
11	-	0.1 ppm (Acetaldehyde)	
12		0.6 ppm (Acetaldehyde)	
13		0.1 ppm (Formaldehyde)	
14		0.6 ppm (Formaldehyde)	

**Table 2:** Second order kinetic constants for the ozonolysis of α-pinene and limonene.

	Pseudo second order kinetic constant for α-pinene [cm^3^ molecules^−1^ s^−1^]	Pseudo second order kinetic constant for limonene [cm^3^ molecules^−1^ s^−1^]
without coexistent CCs	7.85 × 10^−17^	1.46 × 10^−16^
with 0.1 ppm acetaldehyde	6.96 × 10^−17^	1.41 × 10^−16^
with 0.6 ppm acetaldehyde	7.52 × 10^−17^	1.41 × 10^−16^
with 0.1 ppm formaldehyde	6.60 × 10^−17^	1.44 × 10^−16^
with 0.6 ppm formaldehyde	6.81 × 10^−17^	1.51 × 10^−16^
